# The pPSU Plasmids for Generating DNA Molecular Weight Markers

**DOI:** 10.1038/s41598-017-02693-1

**Published:** 2017-05-26

**Authors:** Ryan C. Henrici, Turner J. Pecen, James L. Johnston, Song Tan

**Affiliations:** 1 Center for Eukaryotic Gene Regulation, Department of Biochemistry and Molecular Biology, The Pennsylvania State University, University Park, PA 16802 USA; 2 Schreyer Honors College, The Pennsylvania State University, University Park, PA 16802 USA; 3 Department of Immunology and Infection, Faculty of Infectious and Tropical Diseases, London School of Hygiene and Tropical Medicine, London, WC1E 7HT United Kingdom

## Abstract

Visualizing nucleic acids by gel electrophoresis is one of the most common techniques in molecular biology, and reference molecular weight markers or ladders are commonly used for size estimation. We have created the pPSU1 & pPSU2 pair of molecular weight marker plasmids which produce both 100 bp and 1 kb DNA ladders when digested with two common restriction enzymes. The 100 bp ladder fragments have been optimized to migrate appropriately on both agarose and native polyacrylamide, unlike many currently available DNA ladders. Sufficient plasmid DNA can be isolated from 100 ml *E*. *coli* cultures for the two plasmids to produce 100 bp or 1 kb ladders for 1000 gels. As such, the pPSU1 and pPSU2 plasmids provide reference fragments from 50 to 10000 bp at a fraction of the cost of commercial DNA ladders. The pPSU1 and pPSU2 plasmids are available without licensing restrictions to nonprofit academic users, affording freely available high-quality, low-cost molecular weight standards for molecular biology applications.

## Introduction

DNA molecular weight markers, also known as DNA ladders, are among the most ubiquitous reagents in molecular biology. They permit important estimates of nucleic acid fragment sizes in a wide variety of experiments including restriction enzyme digests, PCR amplifications, Northern and Southern blotting, and just about any application that utilizes native gel electrophoresis. Because the spectrum of fragment sizes in these experiments spans from tens to thousands of base pairs, two or more size ranges of molecular weight standards are often employed. Typical 100 bp ladders provide fragment increments from 100 bp to 1000 bp, and 1 kb ladders span five hundred to several thousand base pairs.

Both 100 bp and 1 kb ladders can either be purchased from commercial sources or generated in the laboratory. The latter includes the traditional EcoRI-HindIII double digestion of lambda phage DNA, and MspI digestion of pBR322 plasmid DNA. DNA molecular weight markers have also been generated by digestion of custom plasmids^1, 2^, by PCR amplification^3–6^, combining plasmid digestion and PCR amplification^7, 8^, and by partial digestion of custom plasmids or genomic DNA^9–11^. Both commercial and homemade sources of DNA molecular weight markers carry advantages and disadvantages. Commercial markers offer a convenient alternative to homemade preparations. The commercial markers provide reference fragments in standard increments and are usually prepackaged in quality-controlled concentrations, allowing quantitation of experimental samples by visual comparison. However, this convenience comes at a financial cost that cannot always be justified given the increasingly limited access to funding worldwide, especially in developing nations. In our laboratory, we have found it more and more challenging to find an inexpensive source of lambda phage DNA for generating the EcoRI-HindIII molecular weight markers we had relied on.

Here we present the pPSU1 and pPSU2 pair of molecular weight marker plasmids that overcome many of the drawbacks of standard commercial and homemade DNA reference ladders. When digested with either PstI or EcoRV, our two plasmids produce 100 bp or 1 kb ladder fragments, respectively. The pPSU plasmids provide a simple, inexpensive method of generating high quality DNA molecular weight markers.

## Results

### Design Considerations

When designing our 100 bp and 1 kb DNA molecular weight markers, our goal was to create a system that was affordable, robust and freely available. We considered using PCR to generate the DNA molecular weight fragments but concluded that the necessary amplification reactions to accomplish two separate and comprehensive sets of ladders would be too complex and too expensive. Instead, we decided to use plasmids as the source of DNA and to generate the molecular weight fragments by complete restriction digestion, avoiding less reliable partial digestions.

With this in mind, we selected the pUC9 cloning vector as the vector backbone of the molecular weight plasmids for two major reasons. First, pUC9 is a high copy number plasmid and relatively large amounts of plasmid DNA can be isolated from standard *E*. *coli* cell cultures. Second, to the best of our knowledge, pUC9 is free of licensing restrictions that would limit distribution of progeny plasmids. Since we hope to make our molecular weight marker plasmids available without licensing restrictions, any patent or intellectual ownership restrictions on the vector or insert fragment sequences would limit this ability. For this reason, we also chose lambda phage DNA as the initial source of the insert DNA to generate the molecular weight ladder fragments. Furthermore, lambda phage genomic DNA has long been used to produce DNA molecular weight markers^12^.

**Figure 1:**
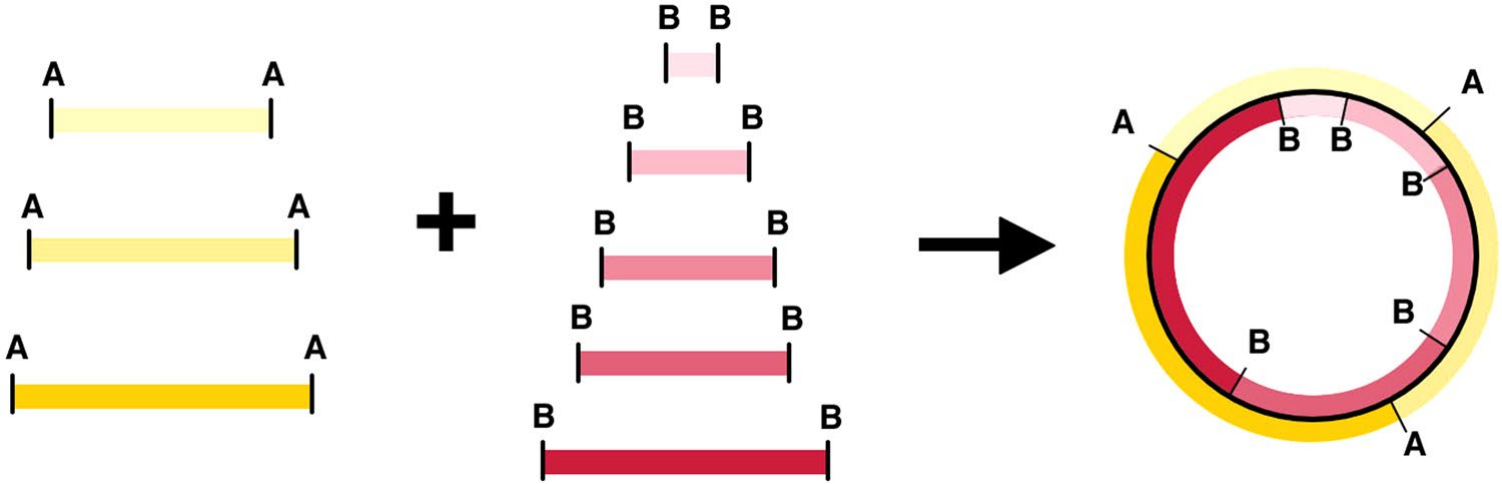
Nesting approach to designing molecular weight marker plasmids. Two distinct sets of ladder fragments delineated by restriction sites A and B can be nested together to form a single, compact plasmid. We implemented this approach using PCR primers to integrate restriction sites into amplified fragments from the source DNA.

We wanted the “100 bp” ladder to contain 100 to 1000 bp fragments in 100 bp increments, and the “1 kb” ladder to minimally provide 1000 to 5000 bp fragments in 1000 bp increments. A single plasmid that sourced all these fragments would need to be approximately 20 kb, and would consequently be difficult to maintain and difficult to produce in high yields. We have instead devised a scheme using a pair of dual purpose plasmids, producing either 100 bp or 1 kb ladder fragments depending on which restriction enzyme is used. The conceptual basis of the dual purpose plasmid is illustrated in Fig. 1. To release the 100 bp and 1 kb ladder fragments from the plasmids, we have selected the restriction enzyme PstI and EcoRV, respectively. These restriction enzymes were chosen because of their commercial availability at relatively low costs and their robustness in restriction digests.

### Overall Design

**Figure 2:**
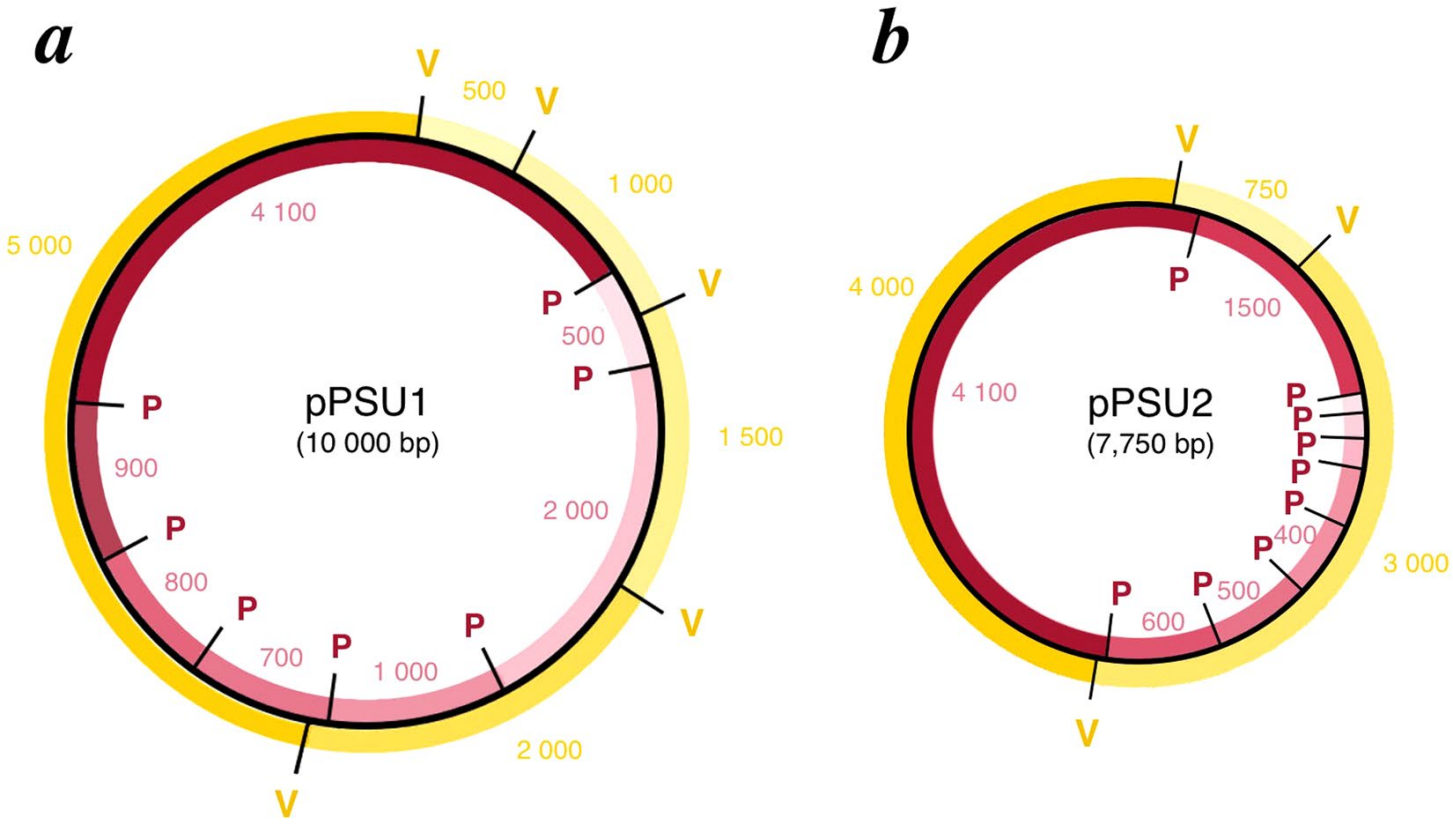
pPSU1 and pPSU2 plasmid maps. Using the nesting approach, nearly 64 kb of 100 bp and 1 kb ladder fragments are incorporated in two high-copy plasmids, pPSU1 (10 kb) and pPSU2 (7.75 kb). The 100 bp ladder fragments produced by PstI digestion of each plasmid are shown in red, and the 1 kb ladder fragments produced by EcoRV digestion are in gold. Restriction sites are denoted by P (PstI) and V (EcoRV). pPSU1 contains the 500, 700, 800, 900, 1000, 2000, and 4100 bp PstI fragments, and 500, 1000, 1500, 2000, 5000 bp EcoRV fragments. pPSU2 contains 50–600, 1500, and 4100 bp PstI fragments, and 750, 3000, and 4000 bp EcoRV fragments. Figure not drawn to scale.

The pPSU DNA molecular weight marker plasmids (pPSU1 and pPSU2) were designed to provide low molecular weight markers when digested with PstI restriction enzyme. In particular, the “100 bp ladder” produced by PstI digestion of both plasmids provides 50, 100, 200, 300, 400, 500, 600, 700, 800, 900, 1000, 1500, 2000, and 4100 bp reference fragments (Table 1 and Fig. 2). The plasmids have also been engineered to produce an additional 500 bp PstI fragment to provide a visual landmark for that fragment on an electrophoresis gel. In contrast, EcoRV digestion of the same two plasmids produces the “1 kb ladder” which provides 500, 750, 1000, 1500, 2000, 3000, 4000, and 5000 bp reference fragments (Table 1 and Fig. 2). Linearizing the 10 kb pPSU1 plasmid and the 7.75 kb pPSU2 plasmid with NcoI (or BglII) provides additional higher molecular weight 7750 and 10000 bp ladder fragments if required.

**Table 1:** DNA ladder fragments from pPSU1 and pPSU2 plasmids.

PstI			EcoRV			EcoRI	linear + EcoRV + EcoRI	linear pPSU1 & pPSU2 + EcoRV pPSU1 & pPSU2
pPSU1	pPSU2	pPSU1 & pPSU2	pPSU1	pPSU2	pPSU1 & pPSU2	pPSU1	pPSU1	
4100	4100	2 × 4100	5000	4000	5000	7001	10000	10000
2000	1500	2000	2000	3000	4000	2999	7001	7750
1000	600	1500	1500	750	3000		5000	5000
900	500	1000	1000		2000		2999	4000
800	400	900	500		1500		2000	3000
700	300	800			1000		1500	2000
500	200	700			750		1000	1500
	100	600			500		500	1000
	50	2 × 500						750
		400						500
		300						
		200						
		100						
		50

### Construction of pPSU1

pPSU1 contains the 500, 1000, 1500, 2000, and 5000 bp EcoRV fragments and the 500, 700, 800, 900, 1000, 2000, and 4100 bp PstI fragments within 10000 total base pairs (Fig. 2a). The plasmid was created in three series of cloning steps involving eighteen plasmids. The majority of cloning steps were performed to replace fragments that migrated anomalously on native polyacrylamide gels.

The first cloning series created the 10 kb pPSU1h plasmid containing the aforementioned EcoRV and PstI fragments. The 500, 1000 and 1500 bp EcoRV fragments were amplified individually, spliced together into a 3000 bp fragment using gene splicing by overlap extension^13^ and cloned into pUC9 (Supplementary Fig. 1, pPSU1a). The 5000 bp EcoRV fragment was created by combining the native pUC9 sequences (origin of replication and ampicillin resistance gene, approx. 2700 bp) with individually amplified 700, 800, and 900 bp PstI fragments assembled end-to-end (Supplementary Fig. 1, pPSU1b). We subcloned the individual 700, 800, and 900 bp PstI ladder fragments sequentially into the pPSU1a vector (Supplementary Fig. 1, pPSU1b) after unsuccessful attempts to splice these fragments together by PCR. Joining the 3′ end of the 1500 bp EcoRV fragment and 5′ end of the 5000 bp EcoRV fragment with an amplified linker completed the 2000 bp EcoRV 1 kb ladder fragment (Supplementary Fig. 1, pPSU1c). The long template QuikChange site-directed mutagenesis technique described by Scott *et al*.^14^. was used to introduce PstI restriction sites within the 1000 bp and 1500 bp EcoRV fragments to create the 500 and 2000 bp PstI 100 bp ladder fragments (Supplementary Fig. 1, pPSU1e). At this point, we determined that the supposed 1500 bp EcoRV fragment was shorter than expected, necessitating steps to replace this defective fragment (Supplementary Fig. 1, pPSU1f, pPSU1g & pPSU1h).

**Figure 3:**
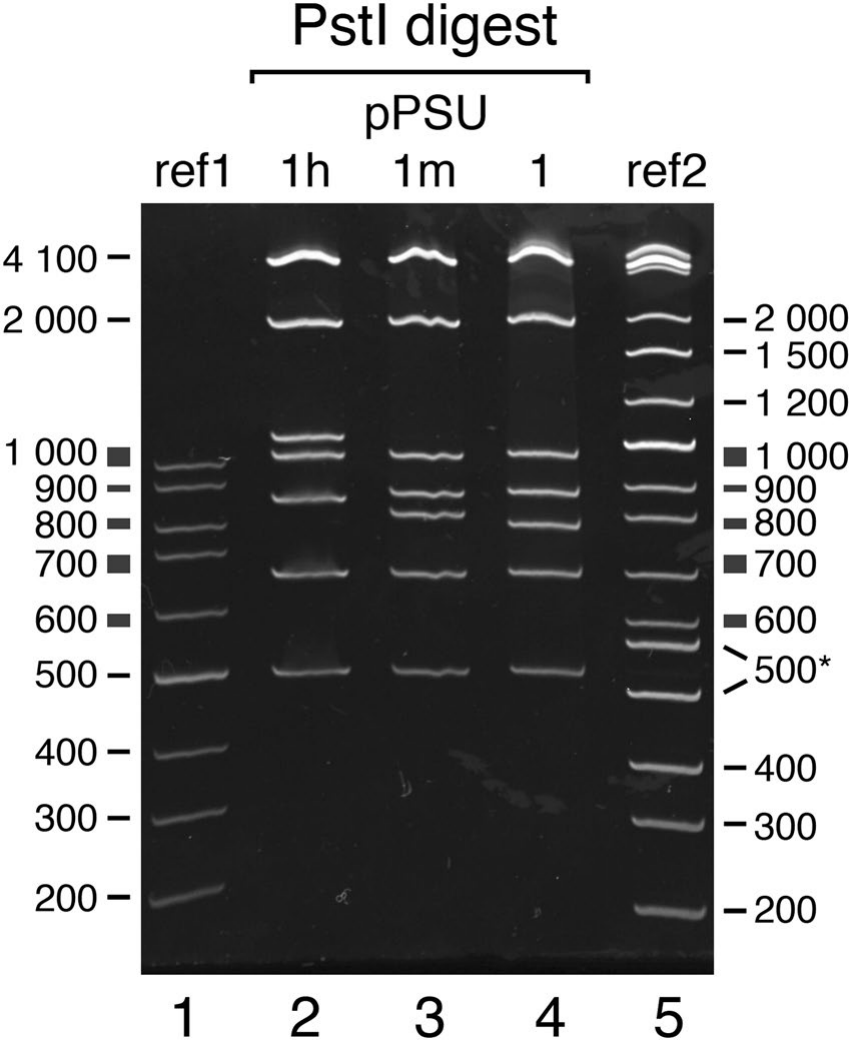
Optimization of 100 bp ladder fragments for polyacrylamide gel electrophoresis (10% acrylamide gel). Lane 1: reference Thermo Scientific Gene Ruler 100 bp ladder (ref1). Lane 2: PstI digestion of the intermediate pPSU1h plasmid contains 800, 900, and 1000 bp lambda DNA fragments which migrate anomalously slowly. Lane 3: pPSU1m contains replacements for the 800, 900 bp fragments from lambda DNA and the 1000 bp fragment from the human Bmi1 RING domain gene. The replacement 800 bp PstI fragment still migrates anomalously slowly. Lane 4: PstI digestion of the final pPSU1 plasmid containing a replacement 800 bp fragment from the human G9a histone methyltransferase gene. Lane 5: reference New England Biolabs 2-Log DNA ladder (ref2). The reference Thermo Scientific Gene Ruler 700 and 1000 bp fragments appear to migrate anomalously, as do the NEB 2-Log DNA ladder 500, 517, 800 and 1000 bp fragments on this 10% acrylamide gel.

Although the pPSU1h plasmid produced the expected EcoRV and PstI fragments that migrated appropriately on agarose gels, we noticed that the 800, 900 and 1000 bp PstI fragments migrated anomalously slowly on 10% acrylamide gels (Fig. 3, lane 2). It is probable that these fragments contained curved DNA with retarded mobility in polyacrylamide gels^15^. For each of these fragments, we designed primers to amplify alternative DNA fragments from the lambda phage genome, examined these amplified fragments on 10% acrylamide gels, and selected fragments that migrated similarly to reference commercial markers. Since we were unable to find three separate lambda DNA fragments that migrated appropriately and lacked confounding restriction sites, we also examined PCR products previously prepared in our laboratory and identified a 1000 bp segment of the RING domain human Bmi1 gene. Substituting the 800, 900 and 1000 bp fragments required five cloning steps resulting in pPSU1m (Supplementary Fig. 2) because our original cloning design scheme had not anticipated the need to replace individual fragments.

Careful examination of the PstI digest fragments of pPSU1m, such as that shown in Fig. 3, indicated that while the replacement 900 and 1000 bp PstI fragments migrated close to what was expected, the replacement 800 bp PstI fragment still migrated anomalously slowly. We employed the same approach used with the 1000 bp Bmi1 gene fragment to identify an 800 bp fragment of the human G9a human methyltransferase gene that appeared to migrate appropriately. A series of three cloning steps replaced the 800 bp fragment in pPSU1m, resulting in the final pPSU1 plasmid (Supplementary Fig. 3).

**Figure 4:**
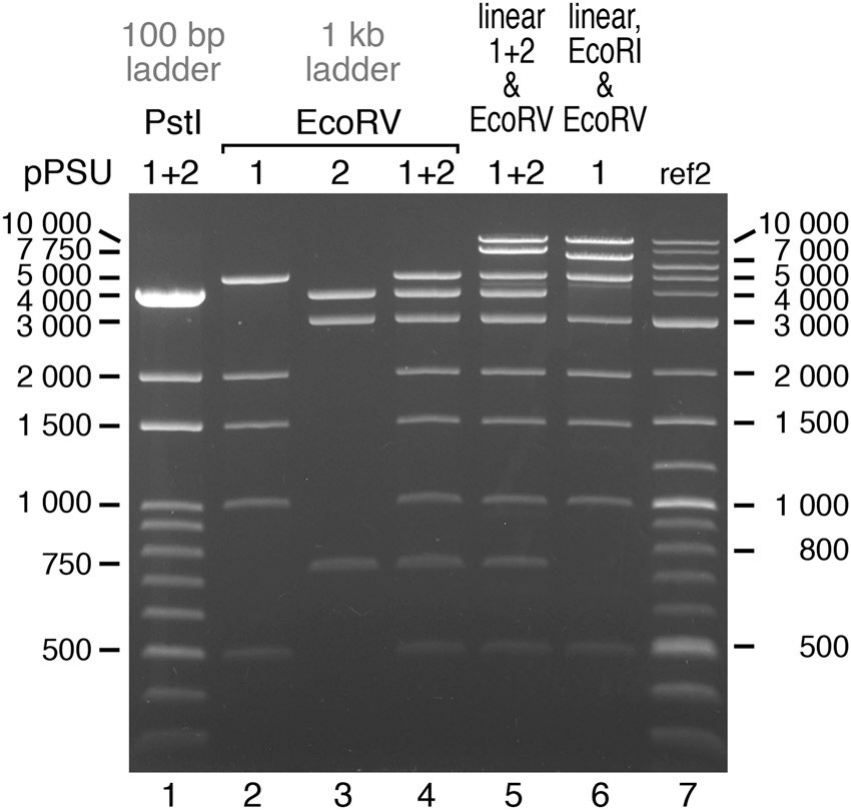
Penn State DNA ladders on agarose gel. The 100 bp (PstI) and 1 kb (EcoRV) Penn State DNA ladders are shown together with the New England Biolabs 2-Log DNA ladder on a 1% agarose gel. Lane 1: 100 bp marker created by PstI digestion of the pPSU plasmids. Lanes 2–4: EcoRV digests of pPSU1 and pPSU2 separately and combined to produce the standard 1 kb Penn State MW Marker. Lane 5: Expanded 1 kb ladder produced by combining the standard 1 kb ladder from lane 4 with NcoI-linearized pPSU1 and pPSU2 to provide additional 7750 and 10000 bp fragments. Lane 6: The 1 kb ladder produced from pPSU1 alone by combining NcoI, EcoRI and EcoRV digests. The faint band migrating between 4000 and 5000 bp in lanes 5 and 6 is likely produced by NcoI star activity. Lane 7: New England Biolabs 2-Log DNA ladder (ref2).

Replacing the anomalously migrating 1000 bp PstI lambda fragment with the human Bmi1 gene fragment fortuitously introduces an EcoRI site 3000 bp (2999 bp to be precise) from the unique EcoRI site from the original pUC9 vector. As a consequence, the pPSU1 plasmid alone can produce a respectable 1 kb ladder with 500, 1000, 1500, 2000, 3000, 5000, 7000 and 10000 bp fragments (EcoRV digestion produces the 500, 1000, 1500, 2000, 5000 bp fragments, EcoRI digestion produces the 3000 and 7000 bp fragments, and NcoI or BglII linearizes the plasmid to produce the 10000 bp fragment) (Fig. 4, lane 6).

### Construction of pPSU2

The second plasmid in the pPSU pair, pPSU2, contains the remaining 750, 3000, and 4000 bp EcoRV 1 kb ladder fragments and the 50, 100, 200, 300, 400, 500, 600, 1500, and 4100 bp PstI 100 bp ladder fragments within 7750 total base pairs. pPSU2 was assembled using a scheme similar to the one used to create pPSU1. The 3000 bp EcoRV fragment was created by a nested organization of multiple PstI fragments. The six 100–600 bp PstI ladder steps were individually amplified and successfully assembled into two polynucleotides, 100–200–300 bp and 400-500-600 bp, using overlap extension PCR (Supplementary Fig. 4, pPSU2d and pPSU2e). These two polynucleotides were cloned into the pUC9 multiple cloning site immediately downstream of a 750 bp EcoRV fragment and an amplified linker (3000frag). The 3000frag linker and the PstI fragments combine to form the 3000 bp EcoRV fragment (Supplementary Fig. 4). A second linker (4000frag) joined the 600 bp PstI fragment and the retained pUC9 sequences upstream of the 750 bp EcoRV step to complete the 4000 bp EcoRV fragment. As with pPSU1, a modified QuikChange site-directed mutagenesis PCR was used to add a PstI site in the 4000 bp EcoRV fragment downstream of the 600 bp PstI fragment creating the 1500 bp PstI fragment (Supplementary Fig. 4, pPSU2i). In addition, mutagenesis was performed to introduce another PstI site 50 bp upstream of the 100 bp PstI fragment (Supplementary Fig. 4, pPSU2f). A further five cloning steps were required to replace anomalously migrating 200 and 600 bp PstI fragments with replacement lambda DNA fragments, resulting in the final pPSU2 plasmid.

### Use of the pPSU1 and pPSU2 plasmids to prepare DNA ladders

For preparing what we refer to as the Penn State DNA ladders, the pPSU1 and pPSU2 plasmids can be digested individually with the appropriate restriction enzyme, or they can be combined and digested in one reaction. The former allows one to vary the ratio of pPSU1 to pPSU2 in the final mixture, but this is probably not necessary as long as the two plasmids are properly quantified. We prepare our 1 kb markers with a ratio of 1:0.8 pPSU1:pPSU2 to account for the difference in the size of the plasmids (10000 bp vs 7750 bp) so that the marker fragments are present in equimolar amounts, but a 1:1 ratio is also adequate if equimolar bands are not required. Sample digestion recipes and notes for preparing the Penn State DNA ladders as well as reference gels for the DNA ladders are provided in Supplementary Note 1.

The difference in reagent costs to prepare the Penn State DNA ladders compared to purchasing commercial ladders is significant. We estimate that it costs us about $5 USD to prepare 1000 lanes of the 100 bp Penn State ladders and about $7 to prepare 1000 lanes of the 1 kb Penn State ladders (Table 2). The alternate 1 kb ladder produced by EcoRV, NcoI and EcoRI digest of pPSU1 costs about $5 for 1000 lanes. These costs calculated for homemade alkaline lysis plasmid prep reagents increase to about $21, $31 and $21 for the 100 bp ladder, 1 kb ladder and the alternate 1 kb ladder, respectively when commercial plasmid prep kits are used. In contrast, New England Biolabs offers a 100 bp ladder for $488, a 1 kb ladder for $212 and their combined 100 bp/1 kb 2-Log ladder for $224 (costs for 1000 lanes). Thermo Scientific ladders are similarly priced at $282 for a 1 kb ladder and $466 for a 100 bp ladder (costs for 1000 lanes).

**Table 2:** Comparison of reagent costs to prepare DNA ladders (1000 lanes).

		pPSU1 plasmid^b^	pPSU2 plasmid^b^	EcoRV enzyme^c^	NcoI enzyme^c^	EcoRI enzyme^c^	PstI enzyme^c^	**total**
homemade plasmid prep^a^	1 kb ladder (EcoRV + NcoI)	$3.00	$3.00	$0.60	$0.60			**$7.20**
	1 kb ladder (EcoRV + NcoI + EcoRI)	$4.00		$0.60	$0.60	$0.20		**$5.40**
	100 bp ladder (PstI)	$2.00	$2.00				$1.20	**$5.20**
commercial kit plasmid prep	1 kb ladder (EcoRV + NcoI)	$15.00	$15.00	$0.60	$0.60			**$31.20**
	1 kb ladder (EcoRV + NcoI + EcoRI)	$20.00		$0.60	$0.60	$0.20		**$21.40**
	100 bp ladder (PstI)	$10.00	$10.00				$1.20	**$21.20**
	NEB 1 kb ladder							**$212**
	NEB 100 bp ladder							**$488**
	NEB 2-Log ladder							**$244**
	Thermo Scientific GeneRuler 1 kb ladder							**$282**
	Thermo Scientific GeneRuler 100 bp ladder							**$466**

All costs in US Dollars.

^a^Costs including bacterial growth media and alkaline lysis plasmid prep reagents assuming 100 µg plasmid yield for 100 ml culture.

^b^We estimate $2.00 per our homemade 100 ml plasmid prep and $10 per midi-scale commercial plasmid prep kit.

^c^Enzyme costs based on using New England restriction enzymes.

**Figure 5:**
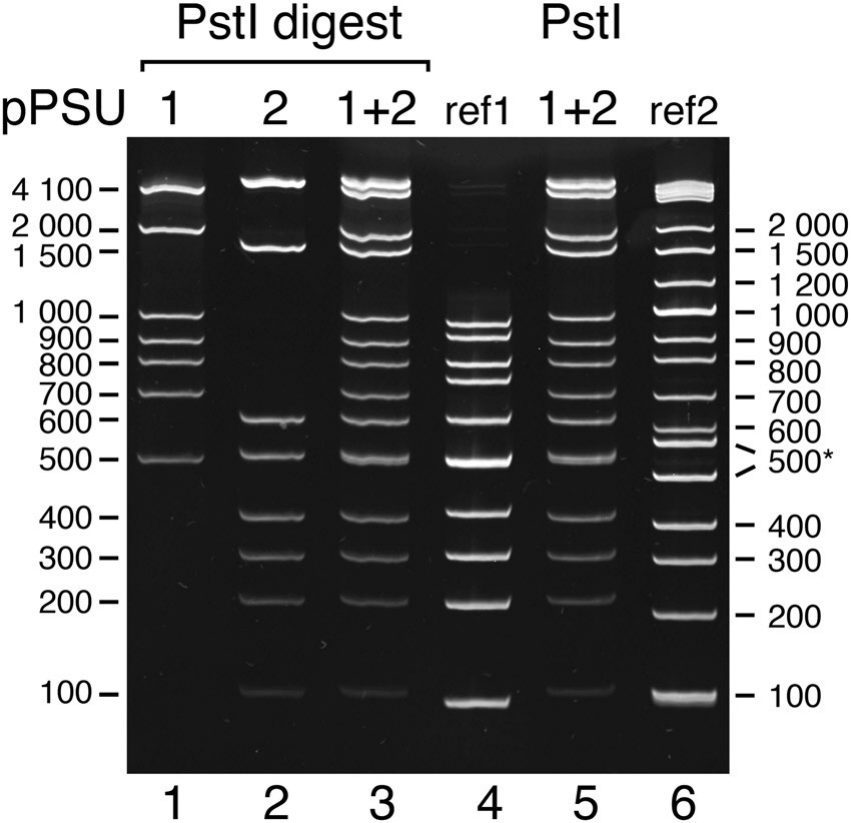
Penn State 100 bp ladder on a polyacrylamide gel (10% acrylamide). Lanes 1–3: PstI digests of pPSU1 and pPSU2 separately and combined to produce the standard Penn State 100 bp ladder. Lanes 4–6: The Penn State 100 bp ladder (lane 5) is compared to the Thermo Scientific Gene Ruler (lane 4, ref1) and NEB 2-log marker (lane 6, ref2). NEB 2-log marker contains 500 and 517 bp fragments to highlight the 500 bp band on agarose gels, but these fragments (marked with *) separate significantly on the 10% acrylamide gel.

Figure 4 shows the behavior of the digested pPSU1 and pPSU2 fragments on a 1% agarose gel. The bands in the 100 bp ladder (PstI digests, lane 1) and in the 1 kb ladder (EcoRV digests, lanes 2–4, and combined with NcoI linearized plasmids, lane 5) migrate as expected, as judged by the same mobility as equivalent sized fragments in the reference ladder (New England Biolabs 2-log DNA ladder). Plots of log(size) as a function of mobility show smooth progressions with no apparent anomalous behavior (all the data points are not expected to fit on a straight line since the all the fragments are not necessarily in the linear fractionation range for the particular concentration of agarose used) (data for the 1% agarose gel is plotted in Fig. 5 and data for a 0.7% agarose gel is plotted in Supplementary Fig. 5).

**Figure 6:**
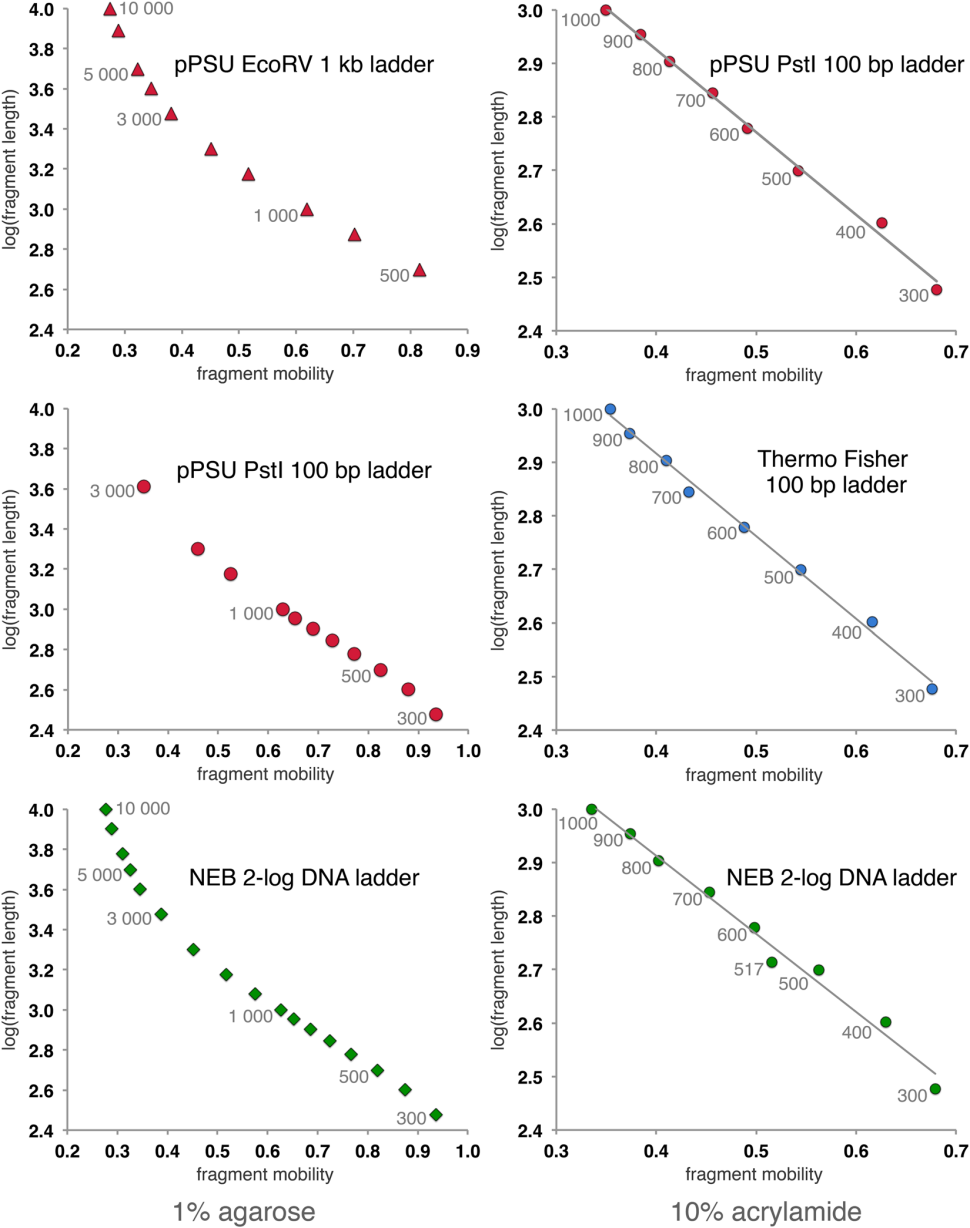
Mobility plots for agarose and acrylamide gels. Log(fragment size) as a function of electrophoretic mobility for the Penn State 100 and 1000 bp DNA ladders, the Thermo Scientific Gene Ruler 100 bp DNA ladder and the New England Biolabs 2-log DNA ladder on 1% agarose and 10% acrylamide gels. Plots are for data shown in Figs 3 and 5.

Although the 100 bp ladder fragments from the PstI digest migrate appropriately on a 1% agarose gel (Figs 4 and 6), the same ladder fragments display more deviations from ideal behavior on a polyacrylamide gel. As noted previously, additional constructs were created to correct anomalously migrating 200, 600, 800, 900 and 1000 bp PstI fragments (see Fig. 3 for the anomalously migrating 200, 600, 800, 900 and 1000 bp PstI fragments). The pPSU1 and pPSU2 plasmids produce 100 bp ladder fragments that migrate close to ideal behavior on a 10% acrylamide gel, with the 400 bp migrating slightly faster than expected (Fig. 5, lanes 2, 3 and 5, Fig. 6). This faster migration is not the result of missing bases from the fragment because the integrity of the fragment was confirmed by DNA sequencing.

## Discussion

We have created the pPSU1 and pPSU2 plasmid pair that produces 100 bp and 1 kb DNA molecular weight reference ladders with similar fragment coverage as commercially available markers at a fraction of the cost. Digestion of the two plasmids with PstI produces a 100 bp ladder between 100 and 1000 bp with additional bands at 50, 1500, 2000 and 4100 bp. The same two plasmids digested with EcoRV produce a 1 kb ladder between 1 and 5 kb with additional bands at 0.5, 0.75 and 1.5 kb. Furthermore, the two plasmids can be linearized with NcoI or BglII to yield additional higher molecular weight 7.75 and 10 kb bands. pPSU1 alone can produce 0.5, 1.0, 1.5, 2.0, 3.0, 5.0, 7.0 and 10.0 kb fragments by combining EcoRV, EcoRI and NcoI digests. The pPSU pair represents a 3.5-fold compression of 63 250 base pairs of molecular weight marker fragments into two manageable plasmids. The coverage from 50 bp to 10 kb will likely satisfy the typical needs for DNA molecular weight markers except when very small or very large DNA molecules are under investigation.

Our ladders are optimized to migrate appropriately on both agarose and acrylamide gels eliminating the need to prepare multiple markers for different experimental applications. We recloned the 200, 600, 800, 900 and 1000 bp PstI bands because the original bands migrated anomalously on polyacrylamide gels. The anomalous migration of DNA molecular weight marker bands on acrylamide gels appears to be reasonably common and also affects the commercial molecular weight markers we used as reference markers during the construction of our DNA molecular weight plasmids. The New England Biolabs 2-Log DNA Ladder contains 500 and 517 bp fragments so that the 500 bp bands will stain more intensely and act as a reference landmark on agarose gels (see for example Fig. 4, lane 7). However, on an acrylamide gel, these bands separate more than expected, complicating interpretation of the reference ladder fragments (Fig. 5, lane 6). The 700, 900 and possibly the 1000 bp bands on the Thermo Scientific GeneRuler 100 bp DNA Ladder also appear to migrate anomalously (Fig. 5, lane 4, Fig. 6).

The pPSU1 and pPSU2 plasmids confer ampicillin resistance and can be grown in standard *E*. *coli* strains and media. We have shown in our laboratory that 100 ml *E*. *coli* cell cultures of pPSU1 and pPSU2 yield at least 100 µg of DNA for each plasmid, enough to produce more than 1000 gel lanes of either 100 bp or 1 kb ladders. We have also cultured 6 liters of the plasmids in Terrific Broth media, and isolated approximately 30 to 60 mg for each plasmid. This suggests that isolating pPSU1 and pPSU2 each from 1 liter of culture can produce 5 to 10 mg of each plasmid, corresponding to 25000 to 50000 gel lanes of both 100 bp and 1 kb ladders.

We would like to offer the pPSU1 & pPSU2 pair of plasmids as an alternative to commercial or other homemade sources of DNA ladders. We therefore make available the pPSU1 and pPSU2 plasmids to the nonprofit academic community without licensing requirements. The pPSU1 and pPSU2 plasmids are being deposited in the Addgene and DNASU repositories to facilitate distribution.

## Methods

### Plasmid construction

Standard molecular biology techniques were used to construct the pPSU1 and pPSU2 plasmids. Oligonucleotide primers used in this work are listed in Supplementary Table 1. The source of the ladder fragments are provided in Supplementary Table 2. Selected ladder fragments were spliced together by overlap extension PCR^13^. Site-directed mutagenesis of plasmids was accomplished using a modified QuikChange mutagenesis procedure^14^. The pPSU1 and pPSU2 plasmids confer ampicillin resistance. All cloning work were performed using the *E*. *coli* TG1 strain^16^. The sequence of the entire pPSU1h and pPSU2 plasmids were confirmed by DNA sequencing, as was the region of the final pPSU1 which replaced the equivalent region in pPSU1h. The sequences of the synthesized dsDNA segments (IDT gBlocks) used to create pPSU1n, pPSU2g and pPSU2j are provided in Supplementary Note 2.

### Gel electrophoresis

1% agarose gels (7 cm long) were electrophoresed in 0.5× TBE buffer (44.5 mM Tris base, 44.5 mM boric acid, 1.25 mM EDTA) at 80 V for 70 minutes. 10% acrylamide gels (7 cm long) contained 40:1 acrylamide:bis-acrylamide, corresponding to 10.25% total monomer concentration (T) and 2.43% crosslinker (C). The acrylamide gels were electrophoresed in 0.5 × TBE buffer at 10 W for 16 min (~400 V).

### Preparation of Penn State DNA ladders

Instructions and notes for preparing the Penn State 1 kb and 100 bp DNA ladders from the pPSU1 and pPSU2 plasmids are provided in Supplementary Note 1. Supplementary Note 1 also includes reference figures for the DNA ladders using data taken from Figs 4 and 5.

## Electronic supplementary material

Supplementary Info
