# Supplementary material for: The pPSU Plasmids for Generating DNA Molecular Weight Markers
Source: Sci Rep. 2017 May 26;7:2438. doi: 10.1038/s41598-017-02693-1 (PMC5446395; doi:10.1038/s41598-017-02693-1)
Supplement: Supplementary file 1 — Supplementary Info [file 41598_2017_2693_MOESM1_ESM.pdf]

The pPSU Plasmids for Generating DNA Molecular Weight Markers

Supplementary Information

Ryan C. Henrici, Turner J. Pecen, James L. Johnston and Song Tan

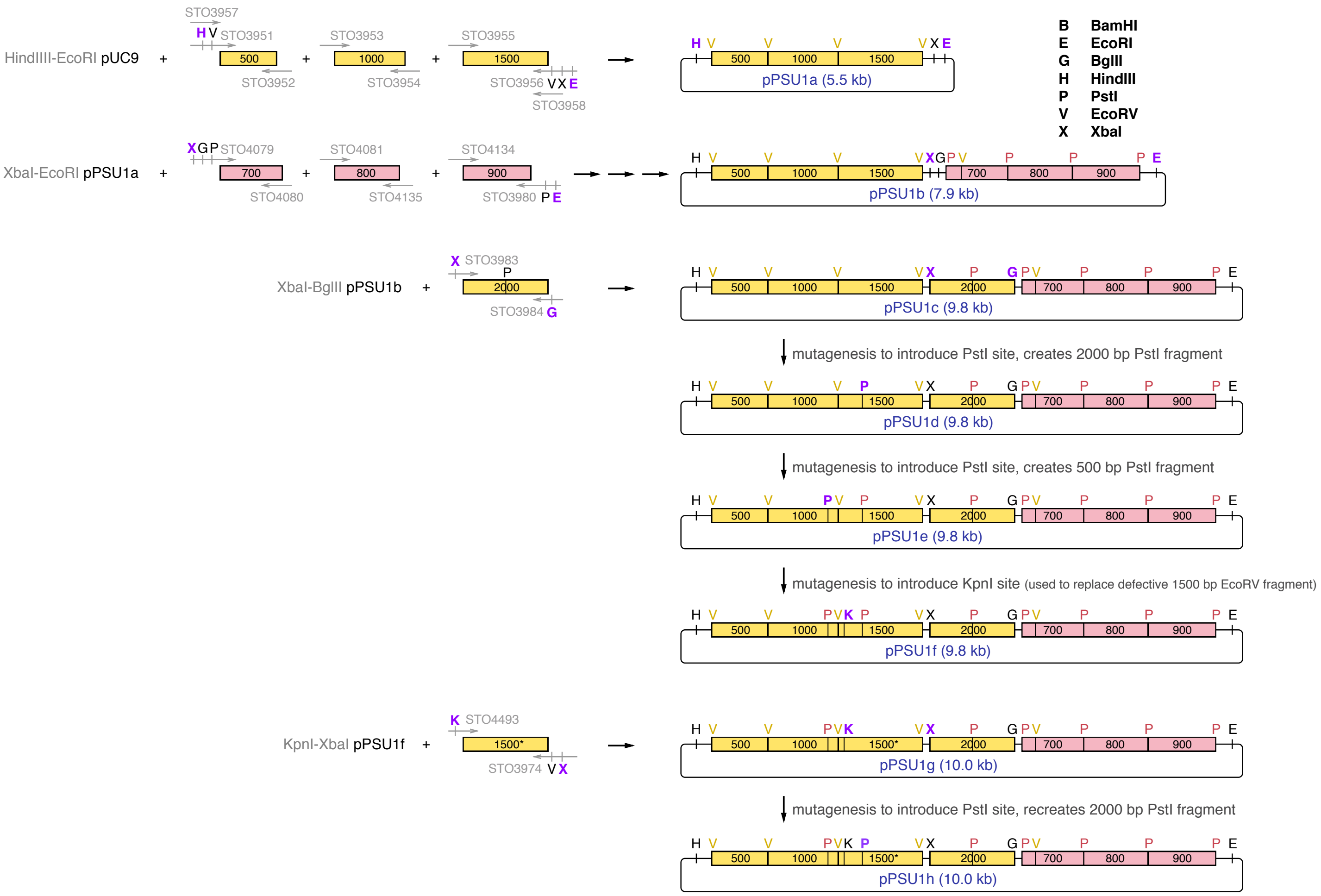

Supplementary Figure 1: Cloning scheme for pPSU1, part 1

Cloned EcoRV fragments are depicted in yellow, and PstI fragments are in pink. Single letter abbreviations for restriction enzyme sites are shown in the upper right. Enzyme sites involved in particular cloning steps are highlighted in bold and in purple. The primers used to amplify individual fragments are shown in the scheme and are detailed in the full list of the primers in Supplemental Table 1. The original 1 500 bp EcoRV fragment was found to contain a deletion, requiring exchange with a replacement 1 500 bp fragment indicated in the scheme as 1500*. Figure not drawn to scale.

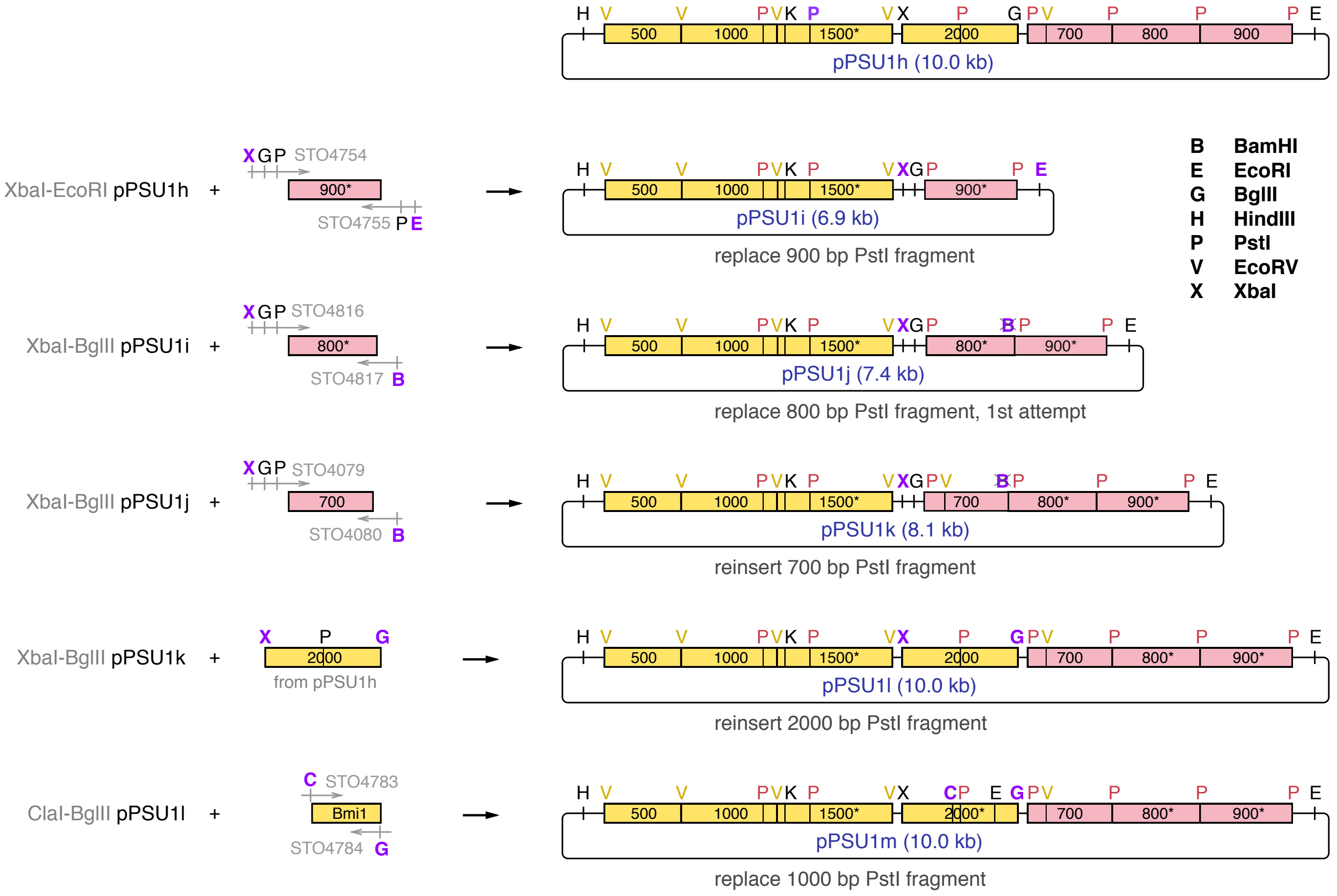

Supplementary Figure 2: Cloning scheme for pPSU1, part 2

Same abbreviations and color coding as for Supplemental Fig. 1. The steps shown replace the anomalously migrating original 800, 900 and 1 000 bp PstI lambda fragments with new lambda fragments (800, 900 bp) or from the human Bmi1 gene (1 000 bp). The BglII end ligated to the compatible BamHI end eliminates both BglII and BamHI sites, shown in the diagram as a crossed-out B.

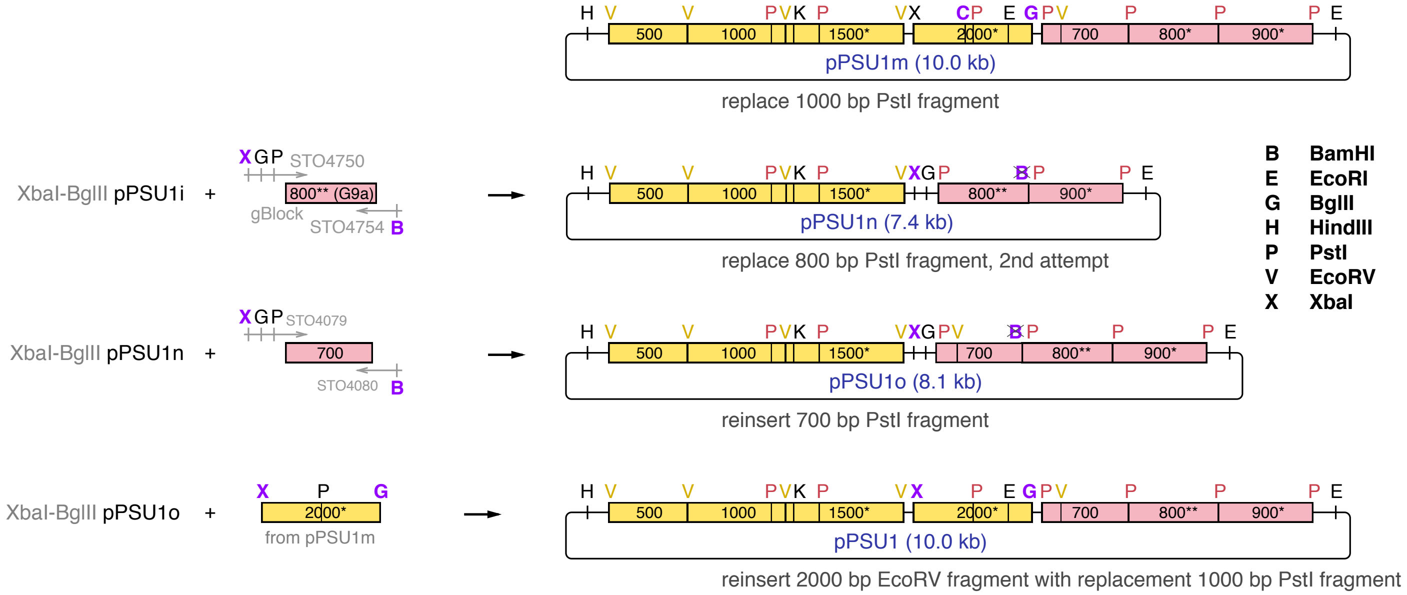

Supplementary Figure 3: Cloning scheme for pPSU1, part 3

Same abbreviations and color coding as for Supplemental Fig. 1. The steps shown replace the anomalously migrating replacement 800 bp PstI lambda fragments with a segment from the human G9a gene.

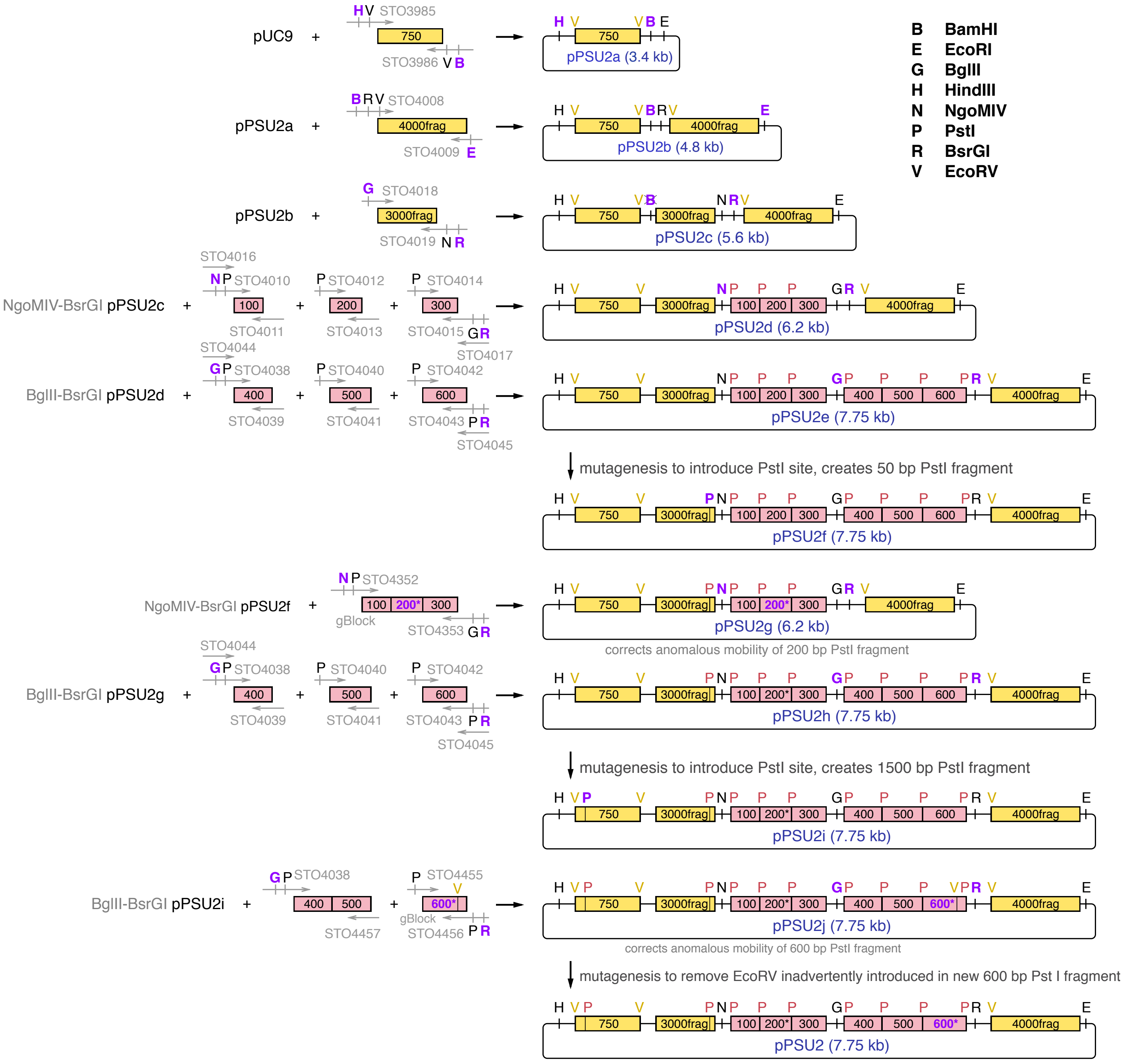

Supplementary Figure 4: Cloning scheme for pPSU2

Same abbreviations and color coding as for Supplemental Fig. 1.

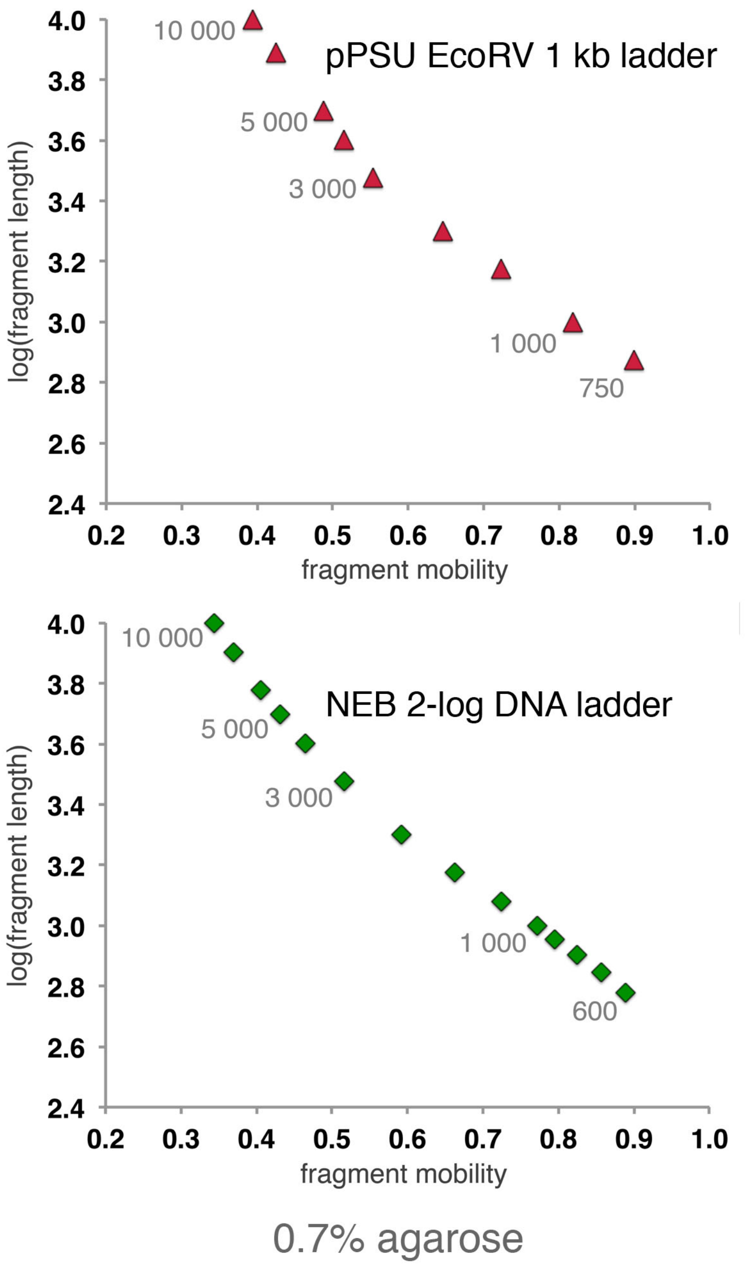

Supplemental Figure 5: Mobility plots for 0.7% agarose gel
Log(fragment size) as a function of electrophoretic mobility for the Penn State 1 kb DNA and the New England Biolabs 2-log DNA ladder on 0.7% agarose gel.

Supplementary Note 1

Preparation of Penn State DNA ladders

1. analytical digest (each plasmid at ~0.1 μg/μl):

water	35.5 μl	water	35 μl
10x NEBuffer 3.1	5 μl	10x NEBuffer 3.1	5 μl
1 μg/μl pPSU1	5 μl	1 μg/μl pPSU1	5 μl
1 μg/μl pPSU2	4 μl	1 μg/μl pPSU2	4 μl
20 units/μl EcoRV	0.5 μl	20 units/μl PstI	1 μl
	50 μl		50 μl

water	17.5 μl
10x NEBuffer 3.1	2.5 μl
1 μg/μl pPSU1	2.5 μl
1 μg/μl pPSU2	2 μl
10 units/μl NcoI	0.5 μl
	25 μl

digest at 37°C for 2 hours, check 1 μl of digest on 1% agarose gel

2. preparative digest (each plasmid at ~0.25 μg/μl):

water	89 μl	water	85 μl
10x NEBuffer 3.1	20 μl	10x NEBuffer 3.1	20 μl
1 μg/μl pPSU1	50 μl	1 μg/μl pPSU1	50 μl
1 μg/μl pPSU2	40 μl	1 μg/μl pPSU2	40 μl
20 units/μl EcoRV	1 μl	20 units/μl PstI	5 μl
	200 μl		200 μl

water	44.5 μl
10x NEBuffer 3.1	10 μl
1 μg/μl pPSU1	25 μl
1 μg/μl pPSU2	20 μl
10 units/μl NcoI	0.5 μl
	100 μl

digest at 37°C overnight, check 0.5 μl of digest on 1% agarose gel

3. dilution of preparative EcoRV digest for ~20 ng/μl 1 kb ladder working stock (-NcoI digest):

10 mM Tris-Cl pH 8.0, 0.1 mM EDTA	1.98 ml
EcoRV digest containing ~0.25 μg/μl pPSU1 and ~0.20 μg/μl pPSU2	0.1 ml
6x gel loading buffer	0.42 ml
	2.5 ml

4. dilution of preparative EcoRV digest for ~30 ng/μl 1 kb ladder working stock (+NcoI digest):

10 mM Tris-Cl pH 8.0, 0.1 mM EDTA	1.93 ml
EcoRV digest containing ~0.25 μg/μl pPSU1 and ~0.20 μg/μl pPSU2	0.1 ml
NcoI digest containing ~0.25 μg/μl pPSU1 and ~0.20 μg/μl pPSU2	0.05 ml
6x gel loading buffer	0.42 ml
	2.5 ml

5. dilution of preparative PstI digest for ~20 ng/μl 100 bp ladder working stock:

10 mM Tris-Cl pH 8.0, 0.1 mM EDTA	1.98 ml
PstI digest containing ~0.25 μg/μl pPSU1 and ~0.20 μg/μl pPSU2	0.1 ml
6x gel loading buffer	0.42 ml
	2.5 ml

Notes

1. Separate restriction digestions of pPSU1 and pPSU2 are recommended if the concentrations of the DNA concentrations of the two plasmids are not well determined, as might be the case if RNA contamination is present.

2. The amount of restriction enzyme provided is a guide. More restriction enzyme may be necessary depending on the quality of the plasmid prep and the source of the restriction enzyme.

3. The suggested working stock of ~20 ng/μl is sufficiently concentrated for our needs, but you may wish to use a higher or lower concentration.

4. We use 10 μl of the 1 kb ladders on agarose gels, and 5 μl of the 100 bp ladder on polyacrylamide gels.

5. Gels for the Penn State ladders are provided on p. 3 of this document.

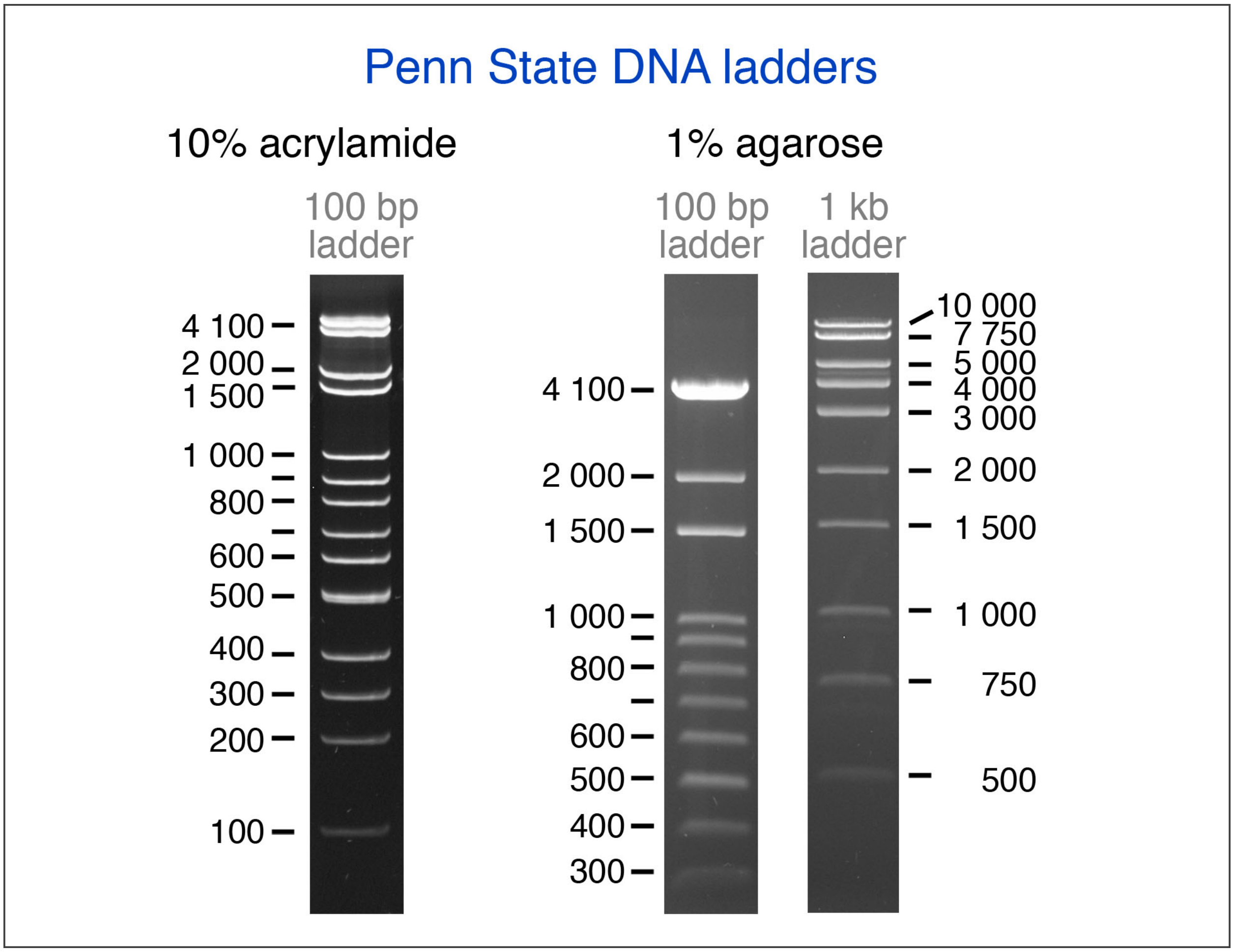

The figure above can be printed, cut along the border and inserted into a 5 inch x 7 inch photo holder.

Supplementary Note 2: Sequences of synthesized fragments

gBlock_100200*300

GCTTCGATGGTCAGAGTTGATCTACGGGGCCGGCCTGCAGGAAGAGGATGGTGCAGCAACCAACAAGAAAACACTGG
CAGATTACGCCCGTGCCTTATCCGGAGAGGATGAATGACGCGACAGGAAGAACTTGCCTGCAGTAACTTTCCCCACA
ACGGAACAACTCTCATTGCATGGGATCATTGGGTACTGTGGGTTTAGTGGTTGTAAAAACACCTGACCGCTATCCCT
GATCAGTTTCTTGAAGGTAAACTCATCACCCCCAAGTCTGGCTATGCAGAAATCACCTGGCTCAACAGCCTGCTCAG
GGTCAACGAGAATTAACATTCCGTCACTGCAGACGGCTCAGGATACGGATAACGGCTACTCCGTGTTTGAGCAGTCA
CTGCTGCGGTATATCGCTGCCGGGCTGGGTGTCTCGTATGAGCAGCTTTCCCGGAATTACGCCCAGATGAGCTACTC
CACGGCACGGGCCAGTGCGAACGAGTCGTGGGCGTACTTTATGGGGCGGCGAAAATTCGTCGCATCCCGTCAGGCGA
GCCAGATGTTTCTGTGCTGGCTGGAAGAGGCCATCGTTCGCCGCGTGGTGACGTTACCTTCAAAAGCGCGCTTCAGT
TTTCAGGAAGCCAGATCTTCAGATCTACTCTGTACACCGAGATCAGC

gBlock_600*

CGGTACGTATCAGGAGCGCCTGAACGCGCTGGCGCAGCAGGCGGATAAATTCGCACAGCAGCAACGGGCAAAACGGG
CCGCCATTGATGCGAAAAGCCGGGGGCTGACTGACCGGCAGGCAGAACGGGAAGCCACGGAACAGCGCCTGAACTGC
AGGGATCCCTTCTGGGGCCGGACGGCATGACATCGCTGCGCGAATATGCCGGTTATCACGGCGGTGGCAGCGGATTT
GGAGGGCAGTTGCGGTCGTGGAACCCACCGAGTGAAAGTGTGGATGCAGCCCTGTTGCCCAACTTTACCCGTGGCAA
TGCCCGCGCAGACGATCTGGTACGCAATAACGGCTATGCCGCCAACGCCATCCAGCTGCATCAGGATCATATCGTCG
GGTCTTTTTTCCGGCTCAGTCATCGCCCAAGCTGGCGCTATCTGGGCATCGGGGAGGAAGAAGCCCGTGCCTTTTCC
CGCGAGGTTGAAGCGGCATGGAAAGAGTTTGCCGAGGATGACTGCTGCTGCATTGACGTTGAGCGAAAACGCACGTT
TACCATGATGATTCGGGAAGGTGTGGCCATGCACGCCTTTAACGGTGAACTGTTCGTTCAGGCCACCTGGGATACCA
GTTCGTCGCGGCTTTTCCGGACACAGTTCCGGATGGTCAGCCCGAAGCGCATCAGCAACCCGAACAATACCGGCGAC
AGCCGGAACTGCCGTGCCGGTGTGCAGATTAATGACAGCGGTGCGGCGCTGGGATATCTGCAGTGTACAGGC

gBlock_800**

GCCAGCACCTGGATCTCTGCAGCCATGGAGATCATCTGCCGGGACGTGGCTCGGGGCTATGAGAACGTGCCCATTCC
CTGTGTCAACGGTGTGGATGGGGAGCCCTGCCCTGAGGATTACAAGTACATCTCAGAGAACTGCGAGACGTCCACCA
TGAACATCGATCGCAACATCACCCACTAACAGCACTGCACGTGTGTGGACGACTGCTCTAGCTCCAACTGCCTGTGC
GGCCAGCTCAGCATCCGGTGCTGGTATGACAAGGATGGGCGATTGCTCCAGGAATTTAACAAGATTGAGCCTCCGCT
GATTTTCGAGTGTAACCAGGCGTGCTCATGCTGGAGAAACTGCAAGAACCGGGTCGTACAGAGTGGCATCAAGGTGC
GGCTACAGCTCTACCGAACAGCCAAGATGGGCTGGGGGGTCCGCGCCCTGTAAACCATCCCACAGGGGACCTTCATC
TGCGAGTATGTCGGGGAGCTGATCTCTGATGCTGAGGCTGATGTGAGAGAGGATGATTCTTACCTCTTCGACTTAGA
CAACAAGGATGGAGAGGTGTACTGCATAGATGCCCGTTACTATGGCAACATCAGCCGCTTCATCAACCACCTGTGTG
ACCCCAACATCATTCCCGTCCGGGTCTTCATGCTGCACCAAGACCTGCGATTTCCACGCATCGCCTTCTTCAGTTCC
CGAGACATCCGGACTGGGGAGGAGCTAGGGTTTGACTATGGCGACCGCTTCTGGGACATCAAAAGCAAATATTTCAC
CTGCCAATGTGGCTCTGAGAAGTGCAAGCACTCAGCCAGGGCCCAGCTGCAGGTGCAGAGT

Supplementary Table 1: Oligonucleotides used

name	sequence	use/comments
STO3951	CGGGGAAGCTTGATATCATCAAAGCCATGAA	forward primer to amplify 500 bp EcoRV lambda fragment
STO3952	GGCTTCGATATCTGGTGAGCGTGTTATCC	reverse primer to amplify 500 bp EcoRV lambda fragment
STO3953	ACACGCTCACCAGATATCGAAGCCTACGC	forward primer to amplify 1 000 bp EcoRV lambda fragment
STO3954	TGGTCTGATATCCATGTACCCGCGTATC	reverse primer to amplify 1 000 bp EcoRV lambda fragment
STO3955	CGCGGGTACATGGATATCAGACCACTTCATTTC	forward primer to amplify 1 500 bp EcoRV lambda fragment
STO3956	CGGAATTCTCTAGAGATATCTAAACATTGCTGATACCGTT	reverse primer to amplify 1 500 bp EcoRV lambda fragment
STO3957	CGGGGAAGCTTGATATC	forward primer to amplify 500 bp+1 000 bp+1 500 bp EcoRV lambda fragments by gene SOEing
STO3958	CGGAATTCGCTCTAGAGA	reverse primer to amplify 500 bp+1 000 bp+1 500 bp EcoRV lambda fragments by gene SOEing
STO3974	CGGAATTCGCTCTAGAGATATCTAAACATTGCTGATACCGTTT	reverse primer to amplify 1 500 bp EcoRV ladder fragment and add 3’ EcoRI, XbaI, and EcoRV sites
STO3980	CGGAATTCCTGCAGTTCAACGACTGCCGC	reverse primer to amplify 900 bp PstI lambda fragment
STO3983	GCCTCTAGACTTCCGATTAGAAACGTCA	forward primer to add linker to complete 2 000 bp EcoRV lambda fragment
STO3984	CGGGAGATCTTAGTCAACACGCACGGT	reverse primer to add linker to complete 2 000 bp EcoRV lambda fragment
STO3985	CGGGGAAGCTTGATATCATCAAAGCCATGAAC	forward primer to amplify 750 bp EcoRV lambda fragment
STO3986	GCGGATCCGATATCGATTCACTGGCTGCAC	reverse primer to amplify 750 bp EcoRV lambda fragment
STO4008	GCGGATCCGCTGTACAGATATCCAAATGAAGCCATAG	forward primer to amplify 4 000 bp EcoRV lambda fragment
STO4009	CGGAATTCTCATAGATGGTCGGTGG	reverse primer to amplify 4 000 bp EcoRV lambda fragment
STO4010	CGGGGCCGGCCTGCAGGAAGAGGATGG	forward primer to amplify 100 bp PstI lambda fragment
STO4011	AAAATCCTGCAGGCAAGTTCTTCCTGTCG	reverse primer to amplify 100 bp PstI lambda fragment with 3’ sequence complementary to 200 bp PstI lambda fragment for gene SOEing
STO4012	GGAAGAACTTGCCTGCAGGATTTTATGTATGAA	forward primer to amplify 200 bp PstI lambda fragment with 5’ sequence complementary to 100 bp PstI lambda fragment for gene SOEing
STO4013	AGCCGTCTGCAGCGTCTGCGCGGG	reverse primer to amplify 200 bp PstI lambda fragment with 5’ sequence complementary to 300 bp PstI lambda fragment for gene SOEing
STO4014	CCCGCGCAGACGCTGCAGACGGCTCAG	forward primer to amplify 300 bp PstI lambda fragment with 5’ sequence complementary to 200 bp PstI lambda fragment for gene SOEing
STO4015	CGGTGTACAGAAGATCTGGCTTCCTGAAAACTGAA	reverse primer to amplify 300 bp PstI lambda fragment
STO4016	CGGGGCCGGCCTGCA	forward primer to amplify 100 bp+200 bp+300 bp PstI lambda fragment gene SOEing product
STO4017	CGGTGTACAGAAGATCTGG	reverse primer to amplify 100 bp+200 bp+300 bp PstI lambda fragment gene SOEing product
STO4018	CGGGAGATCTTTCCGAGCATTTATTAAGC	forward primer to amplify 4 000 bp ladder step linker to complete 3000 bp ladder fragment
STO4019	CGGTGTACAGGGCCGGCTAAAGAACAACCTGACCCA	reverse primer to amplify 4 000 bp ladder step linker to complete 3000 bp ladder fragment
STO4038	CGGGAGATCTCTGCAGGTTGACGGTT	forward primer to amplify 400 bp PstI lambda fragment
STO4039	ATCCGCCTGCAGTTTTTGAGGACTCTGCG	reverse primer to amplify 400 bp PstI lambda fragment with 3’ sequence complementary to 500 bp PstI lambda fragment for gene SOEing
STO4040	AGTCCTCAAAAACTGCAGGCGGATTACA	forward primer to amplify 500 bp PstI lambda fragment with 5’ sequence complementary to 400 bp PstI lambda fragment for gene SOEing
STO4041	AATGCGCTGCAGTTCAGGCGCTGTTCC	reverse primer to amplify 500 bp PstI lambda fragment with 3’ sequence complementary to 600 bp PstI lambda fragment for gene SOEing
STO4042	ACAGCGCCTGAACTGCAGCGCATTGAG	forward primer to amplify 600 bp PstI lambda fragment with 5’ sequence complementary to 500 bp PstI lambda fragment for gene SOEing
STO4043	CGGTGTACACTGCAGGCTCTTCGTCAGCCTC	reverse primer to amplify 600 bp PstI lambda fragment
STO4044	CGGGAGATCTCTGCAGGTT	forward primer to amplify the 400 bp+500 bp+600 bp PstI lambda fragment gene SOEing product
STO4045	CGGTGTACACTGCAGGCTC	reverse primer to amplify the 400 bp+500 bp+600 bp PstI lambda fragment gene SOEing product
STO4079	GCCTCTAGAGCAAGATCTCTGCAGTTGATGCGTGGATGGAGTC	forward primer to amplify 700 bp PstI lambda fragment
STO4080	GCGGATCCAGGTGCTGGCAGGC	reverse primer to amplify 700 bp PstI lambda fragment
STO4081	GCCTCTAGAGCAAGATCTCTGCAGGTGATGATTATCAG	forward primer to amplify 800 bp PstI lambda fragment
STO4134	GCCTCTAGAGCAAGATCTCTGCAGCGCATTGAG	forward primer to amplify 900 bp PstI lambda fragment
STO4135	GCGGATCCTGCTTTATTTAATGGCATCA	reverse primer to amplify 800 bp PstI lambda fragment
STO4245	TGAATGTAACGTAACCTGCAGATCACTGTTGATTCT	forward mutagenesis primer to mutate pPSU-Lambda-A3 to include a 2 000 bp PstI ladder fragment
STO4246	AGAATCAACAGTGATCTGCAGGTTACGTTACATTCA	reverse mutagenesis primer to mutate pPSU-Lambda-A3 to include a 2 000 bp PstI ladder fragment
STO4247	TTTTCGGGGAAATGCTGCAGGAACCCCTATTTGT	forward mutagenesis primer to mutate pPSU-Lambda-A3 to include a 500 bp PstI ladder fragment
STO4248	ACAAATAGGGGTTCCTGCAGCATTTCCCCGAAAA	reverse mutagenesis primer to mutate pPSU-Lambda-A3 to include a 500 bp PstI ladder fragment
STO4249	CATTCTTGAGTCCAACTGCAGAGTATTGTGTACCTT	forward mutagenesis primer to mutate pPSU-Lambda-B5 to include a 50 bp PstI ladder fragment
STO4250	AAGGTACACAATACTCTGCAGTTGGACTCAAGAATG	reverse mutagenesis primer to mutate pPSU-Lambda-B5 to include a 50 bp PstI ladder fragment
STO4352	GCTTCGATGGTCAGAGTTG	forward primer to amplify gBlock_100200*300 PstI fragment
STO4353	GCTGATCTCGGTGTACAGAG	reverse primer to amplify gBlock_100200*300 PstI fragment
STO4416	CAGTCAGGTTAACCTGCAGCGGCATTTTGTCC	forward mutagenesis primer to mutate pPSU-Lambda-B7 to include a 1 500 bp PstI ladder fragment
STO4417	GGACAAAATGCCGCTGCAGGTTAACCTGACTG	reverse mutagenesis primer to mutate pPSU-Lambda-B7 to include a 1 500 bp PstI ladder fragment
STO4455	CGGTACGTATCAGGAGCGCCTGAACGC	forward primer to amplify 600 bp PstI ladder fragment gBlock fragment
STO4456	CGGTGTACACTGCAGATATCCCAGCGCC	reverse primer to amplify 600 bp PstI ladder fragment gBlock fragment
STO4457	CGGTGTACAGTCATGGATCCCTGCAGTTCAGGCGCTGTTC	reverse primer to amplify 500 bp PstI fragment with 3’ restriction sites to enable sequential cloning of replacement 600 bp PstI fragment
STO4467	GTGCGGCGCTGGGACATCTGCAGTGTACAG	forward mutagenesis primer to mutate pPSU-Lambda-B8 to remove accidentally created EcoRV site
STO4468	CTGTACACTGCAGATGTCCCAGCGCCGCAC	reverse mutagenesis primer to mutate pPSU-Lambda-B8 to remove accidentally created EcoRV site
STO4750	GCCTCTAGAGCAAGATCTCTGCAGGCATCAGCGTGGTCTGAG	forward primer to amplify replacement pPSU1 lambda DNA marker 800 bp fragment 2
STO4751	CGCGGATCCGGACTCCTGAAATCTTTACTGC	reverse primer to amplify replacement pPSU1 lambda DNA marker 800 bp fragment 2
STO4754	GCCTCTAGAGCTAAGATCTCTGCAGGTGCAGAGTGATTTGCCGT	forward primer to amplify replacement pPSU1 lambda DNA marker 900 bp fragment 1
STO4755	CTCGGAATTCCTGCAGCGTTATCCGTTGTCTGTATACGG	reverse primer to amplify replacement pPSU1 lambda DNA marker 900 bp fragment 1
STO4783	CGGGATCGATACTGATGTATCATGTCCAAAGAACGATTATCTGCAGATCGAACAACGAGA	forward primer to amplify replacement pPSU1 PstI 1 000 bp fragment (human Bmi1)
STO4784	CGGGAGATCTGATTATGTACATTAACCAGAAGAA	reverse primer to amplify replacement pPSU1 PstI 1 000 bp fragment (human Bmi1)
STO4816	GCCTCTAGAGCAAGATCTCTGCAGCCATGGAGAT	forward primer to amplify replacement pPSU1 PstI 800 bp fragment (human G9a)
STO4817	GCGGATCCGGGCCCTGAGTGCTTGCACTTCTCAGA	reverse primer to amplify replacement pPSU1 PstI 800 bp fragment (human G9a)

Supplemental Table 2: Source of DNA ladder fragments

enzyme	fragment	start	end	sequence	source	plasmid
EcoRV	500	650	1 149	lambda	lambda	pPSU1
	750	650	1 399	lambda	lambda	pPSU2
	1 000	6 681	7 681	lambda	lambda	pPSU1
	1 500	28 223	29 710	lambda	lambda	pPSU1
	2 000	36 060	37 994	lambda	lambda	pPSU1
	3 000	25 345	26 210	lambda	lambda	pPSU2
	4 000	22 948	24 306	lambda	lambda	pPSU2
PstI	50	26 172	26 210	lambda	lambda	pPSU2
	100	2 554	2 656	lambda	synthesized	pPSU2
	200	37 262	37 455	lambda	synthesized	pPSU2
	300	3 856	4 149	lambda	synthesized	pPSU2
	400	19 833	20 232	lambda	lambda	pPSU2
	500	28 223	28 663	lambda	lambda	pPSU1
	500	11 835	12 334	lambda	lambda	pPSU2
	600	2 858	3 447	lambda	synthesized	pPSU2
	700	8 783	9 468	lambda	lambda	pPSU1
	800	2 757	3 535	G9a	synthesized	pPSU1
	900	14 381	15 280	lambda	lambda	pPSU1
	1 000	5	979	Bmi1	cDNA	pPSU1
	1 500	25 340	26 170	lambda	lambda	pPSU2
		743	1 399	lambda	lambda	
	2 000	28 670	29 710	lambda	lambda	pPSU1
		36 060	37 994	lambda	lambda	
	4 100	22 948	24 306	lambda	lambda	pPSU2
